# Medicinal Mushrooms and Their Bioactive Compounds: From Traditional Use to Therapeutic Potential

**DOI:** 10.3390/molecules31101749

**Published:** 2026-05-20

**Authors:** Anna Sadowska, Daria Włosek-Pawełas, Halina Car

**Affiliations:** 1 Department of Experimental Pharmacology, Medical University of Bialystok, Szpitalna 37, 15-295 Bialystok, Poland; 2 Student’s Pharmacological Club, Lazarski University, Świeradowska 43, 02-662 Warsaw, Poland; farmakologiczni@lazarski.edu.pl

**Keywords:** medicinal mushrooms, anticancer activity, bioactive compounds, clinical trials

## Abstract

Medicinal mushrooms have become an important component of modern dietary supplementation and functional nutrition due to their diverse biological activities and long-standing use in traditional medicine. Among the most widely studied and utilized species are *Ganoderma lucidum*, *Lentinula edodes*, *Grifola frondosa*, *Cordyceps militaris*, *Cordyceps sinensis*, *Trametes versicolor*, and *Inonotus obliquus*. Their therapeutic potential is associated with a wide range of biologically active constituents, including polysaccharides, triterpenoids, phenolic compounds, and other secondary metabolites. Experimental and clinical studies indicate that extracts derived from these species may support immune function, modulate inflammatory responses, and exhibit antioxidant, antimicrobial, and anticancer properties. In addition to extensive in vitro and in vivo investigations, a growing number of clinical studies have evaluated the safety and potential therapeutic benefits of medicinal mushroom preparations in humans. In recent years, increasing attention has been directed toward their incorporation into nutraceutical formulations and functional foods aimed at supporting health and preventing chronic diseases. Advances in cultivation technologies and extraction methods have also contributed to improved availability and standardization of mushroom-derived products. This review provides a comprehensive overview of selected medicinal mushroom species commonly used in dietary supplements, focusing on their bioactive constituents, reported biological activities, and potential applications in contemporary medicine.

## 1. Introduction

Approximately 2.2 to 3.8 million fungal species have been identified worldwide, with about 2000 regarded as edible and over 200 recognized for their medicinal properties. Edible mushrooms are typically consumed fresh or dried and are commonly prepared in various culinary forms such as soups, teas, tinctures, or cooked dishes. In contrast, medicinal mushrooms are primarily used in biopharmaceutical applications, often processed into powdered or liquid extracts [1].

Medicinal mushrooms have long been used for the prevention, treatment, and management of various diseases, as well as for supporting overall health and a balanced diet. Their use originates in traditional folk medicine, and their cultivation has long been practiced, with about 20 species currently grown commercially in more than 60 countries. Mushrooms are an important source of biologically active substances, which are estimated to exhibit approximately 130 therapeutic effects, including antitumor, immunomodulatory, cardioprotective, antidiabetic, antioxidant, free radical scavenging, and antiviral activities. Medicinal mushrooms are particularly valued for their high protein content (20–30% of dry matter) and complete profiles of essential amino acids. They are composed of glucans, terpenes, glycoproteins, and peptide- or protein-bound polysaccharides. In addition, they are cholesterol-free, low in total fat, and rich in unsaturated fatty acids. They also contain minerals, amino acids, and several vitamins (e.g., vitamin B1, B2, C, and D) [2,3,4,5,6].

This review aims to provide the therapeutic significance of selected medicinal mushrooms commonly used in dietary supplementation, with emphasis on their historical background, bioactive compounds, biological activities, and clinical potential. It also critically evaluates the current level of evidence and highlights key limitations related to standardization, variability of extracts, and clinical applicability.

## 2. The History of Mushroom Application in Medicine

It is believed that the earliest recorded medicinal use of fungi involved red yeast rice. This practice was documented in China during the 8th century, where the yeast *Monascus purpurea* was cultivated. This yeast produces a bioactive mixture of compounds [7].

The consumption of mushrooms dates back to ancient times, as evidenced by the discovery of edible mushroom remains near archeological sites, such as in Chile, dating back 13,000 years. Notably, a fungus called *Piptoporus betulinus* was found among the belongings of the world’s oldest human mummy, estimated to be about 4000 years old. This fungus is known for its antibacterial and antiparasitic properties [2].

In Chinese culture, lingzhi (*Ganoderma lucidum*) was celebrated as a superior tonic and a symbol of spiritual power and immortality, and was regarded as the “herb of spiritual potency,” associated with success, well-being, divine power, and long life.

Mushrooms have also been an integral component of various ritual practices across numerous regions worldwide. Buddhist monks and early European cultures incorporated them into religious ceremonies as far back as 6000 years ago. There is even a belief that the Buddha died after ingesting a poisonous mushroom called Sukala-maddava. One notable example is the consumption of hallucinogenic mushrooms by members of the ancient Greek elite, including figures such as Socrates and Plato, during the Eleusinian Mysteries—a secretive religious festival [2]. These examples underscore the profound and multifaceted role mushrooms have played across diverse cultures throughout history. Initially used in rituals and nutrition, fungi are now also employed in life-saving medical treatments.

At times, the discovery of mushrooms’ remarkable properties occurred by coincidence. For instance, in the mid-seventeenth century, a melon grower in Paris noticed a fungus sprouting on the remains of a melon. Initially referred to as champignon de Paris, or “Paris mushroom,” it was later identified as *Agaricus bisporus* [8]. This species of mushroom has since undergone clinical trials to evaluate the anticancer activity of its bioactive compounds, particularly in the treatment of breast and prostate cancers [9].

## 3. Selected Medicinal Mushroom Species: From Traditional Use to Preclinical Evidence

A wide variety of mushrooms are documented in traditional medicine and ethnopharmacological records. Among these, *Ganoderma lucidum*, *Trametes versicolor*, *Lentinula edodes*, *Grifola frondosa*, *Inonotus obliquus*, *Cordyceps sinensis*, and *Cordyceps militaris* are considered particularly important due to their long history of use and well-documented biological activity. These medicinal mushrooms are rich sources of bioactive compounds, including polysaccharides, triterpenoids, sterols, phenolic compounds, and other secondary metabolites associated with immunomodulatory, anticancer, antioxidant, anti-inflammatory, antimicrobial, and osteogenic effects. Furthermore, numerous in vitro and in vivo studies have investigated their molecular mechanisms of action, supporting their potential therapeutic applications [10,11].

Search strategies

The literature search for this review was conducted using PubMed, Scopus, Web of Science, and Google Scholar. The search included combinations of keywords related to medicinal mushrooms and their bioactive compounds, including general terms such as “medicinal mushrooms” and “bioactive compounds,” as well as species-specific terms such as *Lentinula edodes*, *Ganoderma lucidum*, *Trametes versicolor*, *Grifola frondosa*, *Inonotus obliquus*, *Cordyceps sinensis*, and *Cordyceps militaris*. Additional keyword combinations addressed biological activities and health effects, including antioxidant, anticancer, antimicrobial, hepatoprotective, cardiovascular, and antidiabetic properties. Furthermore, the reference lists of the selected publications were manually examined to identify any further relevant studies that might have been missed during the electronic database search. After the initial screening, non-English publications were removed. The final dataset comprised 248 sources, including original research articles, review studies, and suitable book chapters.

To retrieve the available published evidence from studies conducted in humans, electronic databases (PubMed, Embase, SCOPUS, ClinicalTrials.gov) were searched for articles published in English. No restrictions were placed on the year of publication. Only completed studies with published results available to date were included.

**Figure 1:**
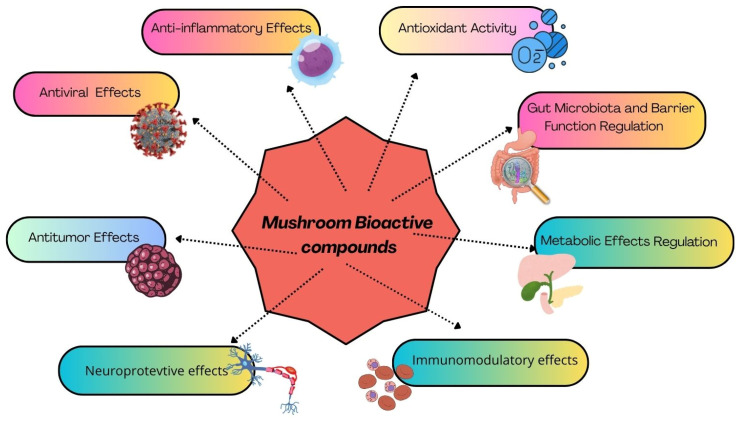
Major biological activities of mushroom-derived bioactive compounds.

Selected bioactive compounds and therapeutic mechanisms of the aforementioned medicinal mushrooms are presented in Table 1, while Figure 1 illustrates the main properties of the bioactive compounds found in medicinal mushrooms.

### 3.1. Trametes versicolor

In traditional Chinese and Japanese medicine, *Trametes versicolor* holds a prominent status as a medicinal mushroom, recognized for its high β-glucan content, which has been shown to stimulate the immune system [138].

The fungus *Trametes versicolor* (L.) Lloyd is commonly referred to as Turkey tail, and it is also known by the Japanese name Kawaratake, which can be translated as “mushroom by the riverbank”. In Chinese, it is referred to as Yunzhi, meaning “cloud mushroom.” It belongs to the Polyporaceae family and is also classified as *Coriolus versicolor*. In ancient Chinese medicine, it was employed to eliminate dampness, reduce phlegm, and treat lung diseases, while Mexican traditional medicine has historically utilized it for the management of ringworm and impetigo [139].

**Figure 2:**
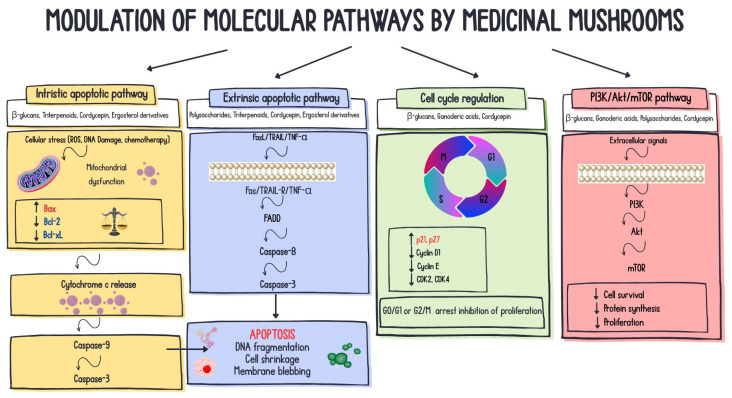
Molecular pathways involved in the anticancer effects of medicinal mushroom-derived compounds. Abbreviations: Bax—Bcl-2-associated X protein; Bcl-2—B-cell lymphoma 2; Bcl-xL—B-cell lymphoma-extra-large; FasL—Fas Ligand; TRAIL—TNF-related apoptosis-inducing ligand; TRAIL-R—TRAIL Receptor; TNF-α—Tumor Necrosis Factor alpha; p21—Cyclin-dependent kinase inhibitor 1 (CDKN1A); p27—Cyclin-dependent kinase inhibitor 1B (CDKN1B); CDK2—Cyclin-Dependent Kinase 2; CDK4—Cyclin-Dependent Kinase 4; PI3K—Phosphoinositide 3-Kinase; Akt—Protein Kinase B (PKB); mTOR—mechanistic Target Of Rapamycin.

Following its discovery in Japan in 1965, the immunomodulatory activity of *Trametes versicolor* led to its incorporation into integrated anticancer treatment regimens for gastric cancer patients in Japan (since 1977) and China (since 1987), commonly alongside radiotherapy or chemotherapy [6,139,140,141]. The fungus contains various compounds; however, the two most extensively studied are polysaccharide peptide (PSP) and polysaccharide K (PSK, Krestin), which are considered the most biologically active components of the mushroom [15,142]. PSP has been observed to induce the release of cytokines, enhance their expression, and also promote the release of chemokines such as TNF-α, interleukins, histamine, and prostaglandin E. It activates natural killer (NK) cells and enhances their infiltration into tumors. This mushroom has been shown to have multiple beneficial effects, including anticancer, antiviral, anti-inflammatory, hepatoprotective, ulcer-protective, and anti-aging properties, as well as memory-improving effects (see Table 1). For three decades, Asian studies have used components of the fungus as supportive therapy for stomach, esophageal, nasopharyngeal, colon, rectal, and lung cancers [13,143]. Interestingly, it has been shown to reduce the adverse effects of chemotherapy and radiation therapy [12,141]. Research has demonstrated its capacity to impede the proliferation of T47D, MCF7, and MDA-MB-231 cells, with a potency comparable to that of mitomycin C, via the induction of apoptosis, upregulation of the genomic guardians, and downregulation of Bcl-2 [31,144]. Recent studies have shown that protein-bound polysaccharides from *C. versicolor* activate the TNF-α/TNFR1 signaling pathway and induce cytotoxicity via the necroptosis mechanism in MCF7 cells [145]. Figure 2 summarizes selected key anticancer molecular mechanisms associated with the activity of bioactive compounds derived from medicinal mushrooms.

Iyekekpolor et al. [146] analyzed methanolic extracts of *Trametes versicolor* (L.) Lloyd to evaluate their nutritional composition, mineral content, anti-inflammatory potential, and possible anticancer activity. The results demonstrated that the mushroom contains a range of essential nutrients, including proteins, carbohydrates, dietary fiber, and low levels of fat, as well as important minerals such as potassium, phosphorus, magnesium, calcium, and trace elements, including iron, zinc, and copper. HPLC analysis of *T. versicolor* extracts revealed the presence of flavonoids such as rutin and kaempferol, together with phenolic compounds that may contribute to their anti-inflammatory and potential anticancer properties. Furthermore, in silico molecular docking analysis indicated that selected bioactive compounds from *T. versicolor* may effectively bind to the HER2 protein, suggesting a potential inhibitory interaction relevant to HER2-positive breast cancer. Overall, the study highlights the potential of *T. versicolor* as a source of nutritionally and pharmacologically valuable bioactive compounds [146].

### 3.2. Ganoderma lucidum

Another highly valued mushroom in China, Japan, Korea, and other Asian countries is *Ganoderma lucidum*, also known as “Ling-zhi” or reishi. In these regions, reishi is referred to as the “mushroom of spiritual powers” and is regarded as a symbol of good health and immortality. In the Far East, in traditional Chinese rituals, it was also used as a talisman for the protection of people and homes against evil [147]. Reishi has a documented history of use in treating various health conditions, including hypertension, diabetes, and insomnia, spanning over 2000 years [148].

This mushroom has been described in the renowned Chinese herbal compendium *Bencao Gangmu*, written by Li Shizhen from the Ming Dynasty. According to this text, the reishi mushroom was consumed by deities to attain immortality, thereby establishing its reputation as “the mushroom of immortality”. The oldest written evidence of reishi, dating back to 25–220 AD, is found in *Shennong Bencao Jing*, which draws on earlier oral knowledge [149].

Nowadays, reishi has emerged as one of the most widely utilized medicinal mushrooms on a global scale. *G. lucidum* is distributed across a wide range of regions, including temperate, tropical, and subtropical areas, such as Europe, America, Africa, India, China, Japan, Korea, and other Asian countries, thriving as a parasite or saprotroph on a broad spectrum of tree species [150].

Reishi has been identified as a potential agent in the treatment of cancer due to the presence of polysaccharides and secondary metabolites, particularly triterpenes such as ganodermic acid, ganoderic acids, ganodermic alcohols, lucidones, and others. These compounds have been shown to possess a variety of biological activities, including hepatoprotective, anti-tumor, anti-proliferative, enzyme-inhibitory, and cytotoxic properties [40,42,43,44,59]. A recent study by Shehzadi et al. [151] analyzed bioactive compounds fractionated from the fruiting bodies of *G. lucidum*, including terpenoids, alkaloids, phenolics, flavonoids, polysaccharides, glycosides, and coumarin derivatives. Fractionation was shown to effectively enrich specific classes of bioactive compounds, with non-polar fractions containing higher concentrations of triterpenoids, whereas polar fractions were enriched in phenolics and flavonoids. Distinct fractions exhibited specific biological activities; for example, Fraction L, rich in phenolic xanthones and carbazole alkaloids, showed strong antioxidant activity comparable to that of ascorbic acid, while Fraction O, enriched in ganoderic acid H and withanolide-related compounds, demonstrated broad-spectrum antimicrobial activity against clinically relevant pathogens. In addition, Fraction B exhibited the strongest α-amylase inhibitory activity, whereas Fraction E showed the highest α-glucosidase inhibition [151].

Polysaccharides, such as α-1,3, β-1,3, and β-1,6-D-glucans, have also demonstrated immune system enhancement and notable anti-angiogenic properties. In addition, studies have revealed that reishi induces apoptosis, inhibits cyclin D1, Bcl-2, and Bcl-xL levels (resulting in cell cycle arrest), and increases Bax and caspase-9 levels in the human breast adenocarcinoma (MCF-7) cell line and in inflammatory breast cancer (IBC) cells [152,153,154]. In Wistar rat models, reishi triterpenes have been shown to significantly reduce the incidence of mammary tumors. Furthermore, the analysis revealed that total triterpenes were associated with a decrease in the mean number of tumors per animal and an extension in the tumor latency period [154].

### 3.3. Lentinula edodes

Shiitake (*Lentinula edodes* (Berk.) Pegler), also known as the “fragrant mushroom” or white flower mushroom, has gained popularity following its widespread use in Asia [139]. It is currently the second most cultivated edible mushroom globally, after *Agaricus bisporus* [144].

Historical records indicate that in the 2nd century AD, the Japanese Emperor Chuai received shiitake as a gift from the indigenous people of Kyushu. Shiitake contains a powerful polysaccharide, lentinan [β-(1→3)-D-glucan], which has demonstrated significant anticancer activity and is approved in Japan as an adjuvant drug for the treatment of gastric cancer [155]. A polysaccharide isolated by Chihara in 1969 exhibited strong antitumor activity in experimental models. In mice bearing Sarcoma 180 tumors, lentinan injections resulted in an approximately 80% tumor reduction or complete regression in most cases. Subsequent studies confirmed its efficacy against allogenic, syngeneic, and autochthonous tumor models. Clinical trials in patients with advanced and recurrent gastric cancer (phase III), as well as colorectal and breast cancers, have reported similarly positive outcomes [69,156,157,158]. The anticancer effects of lentinan have also been confirmed in human cancer cell lines, including HeLa (cervical carcinoma), MCF-7, and T47D (breast cancer), as well as HepG2 (hepatocellular carcinoma) cells [159,160,161,162]. Mechanistic studies indicate that lentinan induces cell cycle arrest by increasing Bax and p21 levels, while downregulating CDK4 and cyclin D1. It also reduces mitochondrial membrane potential, thereby triggering apoptosis, inhibiting cell migration, and promoting autophagy, without affecting normal human cells, including breast cells (HBL-100), hepatocyte cells (LO2), and embryonic kidney cells (293T). Furthermore, in vivo studies demonstrated that lentinan inhibited MCF-7 tumor growth in nude mice by approximately 53%. This effect was associated with modulation of key signaling pathways, including upregulation of p53, phosphorylated ERK1/2, cleaved caspase-3 and PARP, as well as downregulation of NF-κB, Bcl-2, ERα, and telomerase reverse transcriptase (TERT). In addition, suppression of the PI3K/Akt/mTOR pathway contributed to reduced proliferation and enhanced apoptosis in tumor tissues. [161,163]. Collectively, these findings suggest that lentinan acts as a multi-target agent that restores growth control by inducing programmed cell death and inhibiting oncogenic signaling pathways, thereby counteracting the key hallmarks of endocrine-dependent breast cancer. Recent studies have also shown that lentinan exerts significant antitumor activity in breast cancer models in vitro and in vivo by downregulating cancer stem cell markers CD133 and SCGB2A2. In vivo biodistribution analysis confirmed preferential accumulation in tumor tissues, indicating its potential as a targeted therapeutic agent [164].

Lentinan has also demonstrated antiviral activity against infectious hematopoietic necrosis virus (IHNV), inhibiting viral growth by 59.4% at 50 µg/mL and approximately 82.4% at 100 µg/mL in post-addition assays. Additionally, lentinan and its sulfated derivative exhibited activity against tobacco mosaic virus (TMV), causing approximately 56% inactivation at a concentration of 2.5 µg/mL and over 80% at a concentration of 10 µg/mL [165,166].

Eritadenine, a unique compound found in shiitake mushrooms, has been shown to reduce cholesterol levels. However, consumption of undercooked shiitake mushrooms has been associated with shiitake dermatitis, a painful dermatological condition. Shiitake is also a source of antioxidants, including phenolic compounds and ergothioneine. Ergosterol, eritadenine, and β-glucans present in shiitake mushrooms have been demonstrated to possess hypocholesterolemic properties [167,168]. Ergosterol and β-glucans have been shown to reduce cholesterol levels by inhibiting dietary cholesterol absorption and binding bile acids, while eritadenine modulates hepatic phospholipid metabolism, leading to decreased serum cholesterol in animal models of hyperlipidemia [167,168,169]. The observed effects may suggest potential relevance for the prevention and dietary management of hyperlipidemia and obesity-related cardiovascular risk.Shiitake mushrooms exposed to ultraviolet (UV) light synthesize vitamin D2, which has been shown to enhance bone mineral density and improve bone microarchitecture. Research indicates that consumption of vitamin D2-enriched shiitake mushrooms may contribute to skeletal strength and reduce the risk of bone-related disorders, such as osteoporosis. Moreover, Shiitake mushrooms enriched with vitamin D2 exhibit immunomodulatory properties, thereby enhancing the immune response by reducing inflammation and activating immune cells [170,171]. Wiggins et al. [172] demonstrated that vesicle-like nanoparticles derived from *Lentinula edodes* contain a novel lectin (Shictin) with potent antiviral activity against the SARS-CoV-2 Omicron variant. The lectin binds to glycosylated structures on the viral spike protein, thereby inhibiting viral entry into host cells in vitro without inducing significant cytotoxicity. Importantly, viral infectivity was assessed using in vitro cell culture models, including Vero E6 and human ACE2-expressing cells infected with the SARS-CoV-2 Omicron variant [172].

Lentinula edodes produces secondary metabolites, including polyacetylenes and organosulfur compounds, which are low-molecular-weight bioactive constituents. These metabolites exhibit broad antimicrobial activity. The principal polyacetylene, lentinamycin (octa-2,3-diene-5,7-diyne-1-ol), shows strong antibacterial activity against both Gram-positive and Gram-negative bacteria, as well as antifungal effects against a wide range of filamentous fungi and yeasts, with low MIC values indicating high potency, as demonstrated in both in vitro agar diffusion and broth microdilution assays using strains such as *Staphylococcus aureus*, *Escherichia coli*, *Bacillus subtilis*, and *Candida albicans*. Additional polyacetylenes, including octa-3,5,7-triyne-1-ol and nona-triyne derivatives, inhibit spore germination of *Trichoderma* spp. in in vitro co-culture systems, suggesting a role in fungal defense during ecological interactions [173,174].

Organosulfur compounds, particularly lenthionine (1,2,3,5,6-pentathiepane), responsible for the characteristic aroma of shiitake mushrooms, contribute significantly to the antimicrobial profile of *L. edodes*. This compound exhibits antibacterial, antifungal, and anti-yeast activity in vitro, while in vivo studies in mice using a carbon tetrachloride (CCl_4_)-induced liver injury model have demonstrated hepatoprotective effects and inhibition of platelet aggregation. Overall, both polyacetylenes and organosulfur compounds represent key contributors to the antimicrobial and defensive chemical arsenal of shiitake [173,175].

### 3.4. Grifola frondosa

*Grifola frondosa* (Dicks.) Gray (Maitake) is a mushroom species recognized by various names across the world. It is known as the “dancing mushroom” in Japan and as the “Klapperschwamm” in Germany. In other regions, it is identified by names such as the “forest hen,” “sheep’s head,” or the “king of mushrooms.” In China, it is referred to as the “gray tree flower,” and in the West, it is commonly known as Maitake. It is noteworthy that the edibility of the mushroom is limited to its juvenile stage, as it becomes more resilient with age [176].

Maitake is among the most extensively cultivated mushrooms, alongside shiitake and oyster mushrooms. Cultivation in Japan began around 1980, and it is currently widely available in markets worldwide. Its reputation extends beyond its gustatory appeal, encompassing its immune-boosting properties and nutritional value, such as high levels of B vitamins and vitamin D. *Grifola frondosa* is characterized by a pleasant sweet taste and a strong umami flavor, mainly due to its high content of trehalose, glutamic and aspartic acids, and 5′-nucleotides [177]. β-glucans isolated from maitake exhibit immunomodulatory properties by stimulating the production of cytokines and antibodies, thereby contributing to their antitumor effects. The underlying mechanism of its immunomodulatory effect remains to be elucidated. It has been hypothesized that this effect is initiated by a stimulation of neutrophils and monocytes, prompting them to initiate an immune response (see Table 1). In Japan, maitake is employed in the treatment of various ailments, including arthritis, liver inflammation, immune system deficiencies, and cancer. The findings suggest that *G. frondosa* demonstrated higher antitumor activity against breast, liver, and lung cancers, whereas its effects were less pronounced in leukemia, gastric, and bone cancers [178]. Notably, the mushroom has garnered scientific recognition for modulating insulin function and regulating blood sugar levels. In the context of Chinese medicine, maitake is employed to protect the liver, facilitate digestion, enhance immunity, and “moisten the lungs” [139,179].

The β-glucan complex in the mushroom, particularly fraction D, which contains approximately 30% protein, possesses a distinctive structure consisting of a 1,6 chain with 1,3 branches. In addition to the D-fraction, the mushroom contains other bioactive compounds, including Grifolan, X-fraction, MD-fraction, MZ-fraction, and MT-α-glucan. The D-fraction, initially reported by Nanba’s group in the late 1980s [180], has since attracted considerable research attention and has progressively been developed into commercially available complementary and healthcare products. Fraction D has demonstrated the most significant antitumor activity, likely due to its ability to modulate the immune response, exert antiproliferative effects, and induce cytotoxicity of various human cancer cells. Studies have revealed that this particular fraction triggers the release of cytochrome C from mitochondria. This, in turn, has been shown to promote cell dysfunction or apoptosis and to alter the expression of genes involved in cell growth, proliferation, and progression [74,181].

Maitake D-Fraction has been demonstrated to reduce the activity of MMP-2 and MMP-9, which are secreted by triple-negative breast cancer (TNBC) MDA-MB-231 cells and have been definitively associated with the metastatic potential of breast cancer [182,183]. Consequently, D-Fraction could be utilized as an inhibitor of MMP activity to mitigate the invasive potential of TNBC [74]. In the study by Alonso et al. [74], the authors suggested that D-fraction can modify the Bax/Bcl-2 ratio, thereby suppressing pro-survival pathways that are coordinately regulated by PI3K-Akt and ERK. Notably, the D- fraction of maitake, in contrast to other medicinal mushroom extracts, exerts a distinct effect on the prolongation of TNBC cell viability, exhibiting independence from HER2 and hormone receptor status. This characteristic suggests a potential for utilization in the treatment of various subtypes of breast cancer, including both hormone-dependent and hormone-independent, as well as triple-negative types [74].

### 3.5. Inonotus obliquus

Historical evidence indicates that the Khanty people from Western Siberia were among the first to utilize *Inonotus obliquus* (Ach. ex Pers.) Pilát (Chaga) for medicinal purposes, potentially as early as the 12th century. The native Siberians ground Chaga and incorporated it into their daily beverages, soups, and stews. Despite the challenges posed by their inhospitable environment, they ascertained that the regular consumption of Chaga offered a measure of protection against the development of degenerative diseases. This practice has been in continuous use for centuries in Russia and among the Khanty people in Siberia. While it is found across the globe, it is particularly valued in colder environments due to its slow growth rate in such climates. Its unique nutritional profile has recently generated considerable interest. Chaga is a rich source of various components, including polysaccharides, triterpenoids, melanin, and polyphenols, all of which are known to possess anticancer, antioxidant, anti-inflammatory, and other beneficial properties. These components work in synergy to provide a comprehensive array of health benefits [184,185].

The antineoplastic properties of Chaga are due to its ability to induce apoptosis in tumor-transformed cells, as it contains betulinic acid, which has been shown to have cytotoxic effects against various types of cancer [186,187,188]. The cytotoxic potential of 80% ethanolic extracts of *Inonotus obliquus* growing on *Betula pendula* and *Betula pubescens* was evaluated against 31 human cancer cell lines using the sulforhodamine B (SRB) assay. The extracts exhibited moderate cytotoxic activity across all tested cell lines but did not demonstrate high potency (IC_50_ ≤ 20 µg/mL). The strongest growth inhibition was observed for the extract derived from *B. pendula* against HepG2 and CAL-62 cells (IC_50_ = 37.71 and 43.30 µg/mL, respectively), while the extract obtained from *B. pubescens* showed the greatest effect against HepG2 cells (IC_50_ = 49.99 µg/mL). These findings indicate that Chaga extracts display moderate antiproliferative activity, with variability depending on the host tree species [188]. Géry et al. [187] demonstrated that an extract of *Inonotus obliquus* containing betulin, betulinic acid, and inotodiol exerted cytotoxic activity against human lung adenocarcinoma A549 cells. The cytotoxic effect was more pronounced in cancer cells than in normal bronchial epithelial cells, and higher levels of betulin and betulinic acid were associated with stronger antiproliferative activity [187].

Two studies from 1973 and 1981 [189,190] reported that two individuals suffering from psoriasis and stomach ulcer-related pain experienced certain benefits from the consumption of this mushroom. In Eastern European countries such as Russia and Ukraine, this mushroom has a long-standing tradition of use not only for cancer treatment but also for addressing conditions like tuberculosis, stomach ulcers, and heart disease. In Siberia, it is commonly used for the prevention of infections, tuberculosis, and as a remedy for heart, liver, and stomach issues. In the Russian cultural context, it is traditionally prepared as a tea, known as “chaga tea.” Notably, a study has demonstrated that the hot water extract of Chaga exhibited the highest antioxidant activity among various mushrooms [139].

### 3.6. Cordyceps militaris/Cordyceps sinensis

The wild form of *Cordyceps militaris* has been utilized for millennia and is so scarce that it is valued at a higher price than gold. This fungus is held in high esteem and commands a significant price, making it a cherished gift. Historically, the mushroom was exclusively available to royal families, representing a cultural treasure of China. This species is referred to as the “caterpillar mushroom” on account of the mycelial shreds observed growing from the head of a caterpillar. The caterpillar fungus is predominantly found on the Tibetan Plateau, particularly in Tibet, where its exceptionally high economic value allows it to sustain families for extended periods. Consequently, its collection has become an important cultural and economic practice among Tibetan communities in the highlands. Widely used in traditional Chinese medicine, medicinal mushrooms of the *Cordyceps* genus have long been valued for their health-promoting properties. Traditionally, they were used to support the treatment of various ailments, including respiratory disorders such as asthma, as well as serious conditions such as cervical and stomach cancers and viral hepatitis B and C. These traditional applications are consistent with modern findings indicating that *Cordyceps* species exhibit immune-stimulatory, neuroprotective, antimicrobial, anti-inflammatory, and anticancer activities (see details in Table 1) [139,144].

Both *Cordyceps militaris* and its primary active compound, cordycepin, have demonstrated a variety of pharmacological properties, particularly immunostimulatory and antitumor activities. Jeong et al. [191] constructed a *C. militaris* JLM 0636 strain (cordycepin enriched) with 7-fold higher cordycepin content than the wild-type *C. militaris*, which was then administered to CH3/He mice inoculated with FM3A tumor cells. A 30-day feeding period exerted a significant impact on in vivo growth and survival rates of mice. The underlying mechanism of this effect appears to involve increased expression of interferon-γ (IFN- γ) by cytotoxic T cells [191]. The findings further support the antitumor activity of this mushroom, as evidenced by the expansion of cleaved PARP (poly(ADP-ribose) polymerase), cleaved caspase-3, cleaved caspase-8, and Bax expression levels after 14 days of dietary administration [192,193].

The natural *Cordyceps sinensis* is formed when the fungus parasitizes lepidopteran larvae. The fungus invades the larva in autumn and continues to proliferate throughout the winter. By the summer of the following year, the fungal fruiting body emerges from the larva’s head. In Asia, *C. sinensis* has long been regarded as a valuable medicinal material and has been used in traditional Chinese medicine for over 700 years. The fungus occurs naturally in the Tibetan Plateau and nearby areas. The fungus is colloquially termed “chongcao” in China, a shortened version of “dongchong xiacao,” which translates to the Tibetan name “yartsa gunbu” (“summer grass, winter worm”). Polysaccharides are one of the major biologically active components of *C. sinensis*. Due to the high cost of natural *C. sinensis*, cultured mycelium has become an important alternative source of polysaccharides [118,194].

Cordycepin extract from *Cordyceps sinensis* has demonstrated a capacity for antineoplastic activity in human breast cancer (BC) cell lines, including TNBC MDA-MB-231 and MCF7. The observed properties are attributed to the release of cellular lactate dehydrogenase, the disruption of mitochondrial function, and the production of reactive oxygen species (ROS). Furthermore, the fungus has been observed to downregulate Bcl-2, a critical anti-apoptotic protein, and concomitantly increase the levels of pro-apoptotic proteins. Noteworthy are additional activities, including autophagy, DNA damage, and the targeting of cancer cells. The efficacy of cordycepin was verified in a 4T1 murine mammary carcinoma model, where 35-day treatment significantly reduced tumor mass, tumor volume, and the number of metastatic lung colonies [193,195,196]. These results indicate that cordycepin from *Cordyceps sinensis* shows promising preclinical antitumor activity, suggesting potential relevance for further clinical investigation in breast cancer, including TNBC. However, its clinical application remains limited by the lack of human studies.

Studies have demonstrated that *C. sinensis* polysaccharides exhibit antioxidant activity in vitro [197]. Cellular research has shown that the mycelial polysaccharide CME-1 (25–100 μg/mL) protects RAW264.7 cells against hydrogen peroxide-induced oxidative stress by inhibiting sphingomyelinase activity and reducing C16- and C18-ceramide levels [198]. According to Sheu et al. [199], CME-1 polysaccharides suppress the LPS-induced inflammatory response in RAW264.7 cells by inhibiting the activation of the p65, Akt, and MAPK signaling pathways and by upregulating ceramide-induced PP2A activation [199].

It is noteworthy that *Istia sinclairii*, classified within the genus *Cordyceps*, has historically been employed in ancient Chinese medicinal practices as an elixir believed to confer “eternal love.” The metabolite of this fungus is fingolimod, a synthetic derivative of myriocin. This metabolite possesses robust immunosuppressive properties, which led to its approval by the U.S. FDA in September 2010 as a new treatment for multiple sclerosis [7,200].

## 4. Clinical Studies on Selected Medicinal Mushrooms

Medicinal mushrooms offer great potential for the development of innovative functional foods and may also be useful in the prevention and treatment of various human diseases. However, further research is needed to better understand how their bioactive compounds interact with food components and/or drugs.

Table 2 summarizes selected clinical trials investigating the potential of medicinal mushrooms discussed in this review; however, no clinical trials on *Inonotus obliquus* met the established inclusion criteria, despite numerous in vitro and animal studies.

Several studies reported beneficial immunological outcomes, including increased lymphocyte counts, enhanced CD8^+^ T-cell and B-cell responses, elevated NK cell activity, and increased cytokine production such as IFN-γ and IL-2 [201,202,203,204,205,206,207,208]. Modulation of gut microbiota composition, characterized by increased α-diversity and enrichment of beneficial taxa (e.g., *Bacteroides*, *Prevotella*), was also observed [209,210]. In oncology-related contexts, certain mushroom-derived products (e.g., AHCC^®^, lentinan, maitake extracts) were associated with improved immunological parameters, reduced treatment-related adverse effects, improved quality of life, and, in some cases, reduced disease recurrence following curative interventions [204,205,207,211,212,213]. Additional beneficial effects included improvements in lipid profiles and liver enzyme levels [214], enhanced physical performance parameters such as aerobic endurance and walking speed [215], improved mucosal immunity and immune biomarkers [203,216], and clearance of persistent high-risk HPV infections [213,217,218].

Some interventions resulted in statistically significant but clinically modest or transient effects, particularly in immune cell phenotypes or microbiota composition, without consistent translation into clinical outcomes [210,219,220]. In these studies, immunological changes were often dose-dependent, short-lived, or influenced by confounding factors such as antibiotic exposure, limiting their broader clinical relevance [220]. Similarly, improvements in training metrics or selected immune parameters did not consistently translate into enhanced overall performance or functional outcomes [221].

Several studies demonstrated no significant clinical benefit in specific patient populations. These included patients with type 2 diabetes or metabolic syndrome [222], Gulf War Illness [223], rheumatoid arthritis receiving DMARD therapy [224], childhood asthma [225], and mildly hypercholesterolemic adults [210]. In isolated cases, higher doses were associated with worsening of symptoms or unfavorable metabolic effects, such as increased fasting glucose or body weight [216,223]. However, serious adverse events were rare, and overall tolerability remained high across studies [222,223,224,225].

**Table 2:** Clinical research evidence on medicinal mushrooms.

Mushroom Active Compound/Preparation	Participants	Study Design	Administration (Dose, Duration)	Results/Effect	Ref. Clinical Takeaway
*Trametes versicolor* (*Coriolus versicolor*) PSP (polysaccharide peptide)	Healthy volunteers–effect on gut microbiota, 24 healthy volunteers.	Randomized, double-blind, placebo-controlled clinical trial.	A total of 3 g PSP daily for 8 weeks; effects were confirmed by PERMANOVA analysis.	Statistically significant modification of gut microbiota (↑ α-diversity, ↑ *Bacteroides* and *Prevotella*); no adverse effects.	[209] Prebiotic and immunomodulatory effects.
*Trametes versicolor* (*Tv*) (*Coriolus versicolor*) freeze-dried mycelial powder	Breast cancer, 21–75 years female in stage I–III, infiltrating ductal adenocarcinoma of the breast who have undergone surgery and chemotherapy, and are able to begin study treatment within 5 days after the last dose of radiotherapy.	A standard phase 1 dose-escalation study using a 3 + 3 design was conducted to determine the maximum tolerated dose (MTD) of Tv.	The planned dose levels were 3 g, 6 g, 9 g, 12 g, 18 g, and 24 g. Participants were enrolled up to the 9 g cohort only.	Increased lymphocyte, CD8^+^ T cell, and B cell counts; enhanced NK cell activity at higher doses.	[201] Well-tolerated up to 9 g/day; no dose-limiting toxicities. Mild to moderate adverse events only.
*Trametes versicolor* (*Coriolus versicolor*) Multi-ingredient vaginal gel containing *C. versicolor*, *Azadirachta indica*, carboxymethyl-β-glucan, hyaluronic acid, Centella asiatica, Aloe vera, α-glucan oligosaccharide	Women with HPV-related low-grade cervical lesions (LSIL/ASC-US/AGUS), 101 randomized: 59 treatment, 32 control evaluable.	Multicenter, randomized, open-label, controlled trial (watchful waiting as control).	treatment once daily × 21 days, then an alternate-day therapy up to 6 months.	After 6 months, lesion repair (normal cytology + concordant colposcopy) was significantly higher with Papilocare (84.9% vs. 64.5%, *p* = 0.031), especially in HR-HPV women (87.8% vs. 56.0%, *p* = 0.003). After 3 months, early improvement was also greater (78% vs. 54.8%, *p* = 0.023). HPV clearance at 6 months showed a positive trend (59.6% vs. 41.9%, *p* = 0.118), with Scheme B achieving significance (75.9% vs. 41.9%, *p* = 0.008). Cervical re-epithelization scores improved more with Papilocare (4.5 vs. 4.1, *p* = 0.017). Vaginal health and perceived stress showed favorable trends in the treatment group; most adverse events were mild or moderate (vaginal burning, candidiasis).	[217] Papilocare^®^ vaginal gel improved repair of HPV-related low-grade cervical lesions, with trends toward HPV clearance, better re-epithelization, stress reduction, and high adherence.
*Ganoderma lucidum* Ganoderma lucidum spore oil (GLSO)	Dyslipidemia, 110 participants.	Randomized, double-blind, placebo-controlled clinical trial.	12 weeks, 3 g GLSO daily.	↓ triglycerides, ↓ total cholesterol, ↓ LDL; ↑ HDL; improvement in liver enzymes; no adverse events.	[214] Confirms the metabolic effects of GLSO on lipid profile and liver function.
*Ganoderma lucidum* *G. lucidum* powder compared with *Ceratonia siliqua* flour	Women with fibromyalgia, 64 women (32 GL, 32 CS. Final efficacy analysis: 25 GL, 23 CS.	Randomized, double-blind, controlled clinical trial (parallel groups).	6 g/day for 6 weeks	In the *Ganoderma lucidum* group, significant improvements were observed in aerobic endurance (6 min walk test), lower limb flexibility (chair sit-and-reach), and walking velocity (20 m test) compared with *C. siliqua*. No relevant benefits were found in strength, balance, or trunk endurance. In intent-to-treat analysis, only lower limb flexibility remained significant. Adverse effects were mild (nausea, diarrhea, discomfort, nervousness), leading to some withdrawals, but no serious events occurred.	[215] *Ganoderma lucidum* at 6 g/day improved aerobic endurance, flexibility, and walking speed in women with fibromyalgia, suggesting potential benefits for physical fitness.
*Ganoderma lucidum* GL (Hot water/alcohol extract: 12% polysaccharides, 4% triterpenes); Stinging nettle (pure leaf); Epimedium (20% icariin extract)	Gulf War Illness (GWI), 25 men with GWI.	Placebo-controlled, pseudo-randomized, crossover clinical trials.	Tested in sequence: 30 days placebo, 30 days low dose, 30 days high dose. GL: 1600 mg (low dose) or 3200 mg (high dose) daily.	Reishi did not improve GWI symptoms. Compared to placebo, low dose showed no effect (*p* = 0.603), while high dose increased symptom severity (*p* = 0.012). Pain and fatigue did not improve. Overall, reishi was associated with a worsening of symptom severity at higher doses. Adverse events included diarrhea, flushing, headaches, GI upset, worsening fatigue, or GERD, but were generally mild.	[223] Ganoderma lucidum extract did not benefit Gulf War Illness and may worsen symptoms at higher doses.
*Ganoderma lucidum* *with San Miao San* (*SMS*) Capsules with *G. lucidum* extract + SMS (mix of *Rhizoma atractylodis*, *Cotex phellodendri*, *Radix achyranthes*)	Rheumatoid arthritis (active disease despite disease-modifying antirheumatic drugs (DMARDs)), 65 patients (32 treatment, 33 placebo).	Randomized, double-blind, placebo-controlled pilot trial.	For a total of 24 weeks *G. lucidum* (4 g) and SMS (2.4 g) daily.	ACR20 response: 15.6% vs. 9.1% (NS). Pain and patient global scores improved significantly within treatment group but not vs. placebo. No significant effect on tender/swollen joints, physician global, HAQ, ESR, CRP, lymphocyte subsets, or plasma cytokines. Ex vivo IL-18 production decreased significantly in treatment group, but clinical relevance is unclear. No antioxidant effect (FRAP, ascorbic acid unchanged). Adverse events mild (GI upset, insomnia, sweating); 8 in treatment vs. 14 in placebo. No serious adverse events.	[224] Ganoderma lucidum with SMS was safe and may reduce pain perception, but showed no significant anti-inflammatory, antioxidant, or immunomodulatory effects in RA patients on DMARDs.
*Ganoderma lucidum* (lingzhi) Longan syrup (*Dimocarpus longan* Lour) with 0.5% lingzhi mushroom extract	Healthy adults–immune and inflammatory modulation, glycemic safety.	Two parts: 1) Single-dose, randomized crossover (vs. glucose solution, *n* = 20) 2) 12-week, single-group prospective trial (*n* = 8).	For 12 weeks daily intake of 5 mL of syrup.	The syrup produced a lower postprandial glucose excursion than a 50 g glucose solution (iAUC 327.8 vs. 384.1 mg/dL·h, *p* < 0.05), though classified as high glycemic index (GI = 85.35). In the 12-week trial, fasting glucose, HbA1c, liver enzymes, renal markers, immunoglobulins (IgG, IgM, IgA, IgE), and CRP remained stable. Individual-level trends included improved IgG in 75% of participants and reduced IgM in those with high baseline values, plus CRP reduction in one participant with elevated baseline CRP. No adverse events or metabolic harm were observed.	[219] Longan syrup with lingzhi extract was safe, did not worsen glycemia, and showed preliminary immune-modulatory trends.
*Ganoderma lucidum* (Lingzhi/Reishi) ± *Cordyceps sinensis* Ganoderma lucidum extract	Type 2 diabetes mellitus with metabolic syndrome (cardiovascular risk factors), 84 participants (54 intervention, 30 placebo).	Randomized, double-blind, placebo-controlled, parallel-group clinical trial	3 g/day for 16 weeks.	No effect on HbA1c (Δ 0.13%, *p* = 0.60) or fasting plasma glucose (Δ 0.03 mmol/L, *p* = 0.95). No significant differences in BP, triglycerides, waist circumference, BMI, CRP, total cholesterol, LDL, HDL, or ApoA/B. Well-tolerated, mild side effects only; no serious adverse events related to intervention.	[222] *Ganoderma lucidum* (alone or with *Cordyceps*) was safe but ineffective in improving glycaemic control or metabolic syndrome parameters in patients with type 2 diabetes.
*Ganoderma lucidum* (Reishi) + *Lentinula edodes* (Shiitake) + Baker’s yeast Proglucamune^®^:β-glucans blend derived from reishi, shiitake, and yeast	Protective Qi Deficiency (PQD)—a traditional Chinese medicine concept linked to susceptibility to infections (URTI, fatigue, aversion to wind/cold, sweating), 21 adults with PQD.	Single-arm, pre–post proof-of-concept trial with “deceptive blinding” (participants and staff told it was placebo-controlled).	A total of 200 mg/day for 8 weeks (2 tablets daily).	Recruitment (44.7%), compliance (95%), and retention were good. PQD prevalence decreased progressively from 100% at baseline to 9.5% after 8 weeks. A multivariate PQD risk score (low voice/apathy, aversion to wind/cold, Cun pulse) predicted PQD with high accuracy (AUC 0.98). No adverse events were reported.	[226] β-glucan supplementation (from reishi, shiitake, yeast) appeared safe and associated with improvement in PQD symptoms.
*Lentinula edodes* (Shiitake) Active hexose-correlated compound (AHCC): a standardized extract of cultured *L. edodes* mycelia	Hepatocellular carcinoma (HCC) (post-resection), 29 patients post-radical HCC resection,	Single-arm, open-label, no control group.	A total of 1 g AHCC × 3/d for 2 years.	Reported 2-year recurrence-free survival of 48–55%; stabilization of nutrition and inflammation; no adverse events.	[211] AHCC may be safe and effective in preventing HCC recurrence after curative hepatectomy.
*Lentinula edodes* (Shiitake) Active hexose-correlated compound (AHCC^®^), standardized extract of cultured *L. edodes* mycelia (α-glucan-rich)	Persistent high-risk HPV infection (>2 years) in women > 30 years, 50 women (25 AHCC, 25 placebo).	Randomized, double-blind, placebo-controlled phase II clinical trial, with optional unblinded crossover.	AHCC 3 g/day for 6 months followed by 6 months placebo vs. placebo for 12 months; follow-up to 18 months.	After 6 months, 63.6% (14/22) of women on AHCC were HPV RNA/DNA negative, with 64.3% of responders maintaining durable clearance 12 months off treatment. In contrast, only 10.5% (2/19) in the placebo group cleared HPV at 12 months. In placebo participants crossing to AHCC, 50% achieved HPV clearance after 6 months. Overall, 58.8% of all women receiving AHCC cleared HPV. Clearance correlated with suppression of IFN-β below 20 pg/mL, accompanied by increased T lymphocytes and IFN-γ. NK cell levels did not change. AHCC was well-tolerated, with only grade 1, self-limited adverse events (nausea, bloating, mild fatigue).	[213] AHCC^®^ 3 g/day safely promoted clearance of persistent high-risk HPV infections, with durable responses in most cases where IFN-β was suppressed. Provides the first systemic, non-invasive treatment option candidate; phase III studies warranted.
*Lentinula edodes* (Shiitake) Active Hexose Correlated Compound (AHCC^®^), standardized extract of cultured *L. edodes* mycelia	Pancreatic ductal adenocarcinoma (PDAC) (patients on chemotherapy)—prevention of taste disorders and supportive care, 98 patients (55 AHCC^®^, 43 placebo).	Randomized, double-blind, placebo-controlled phase III clinical trial.	A total of 6 g/day for 8–12 weeks during chemotherapy.	In this phase III trial, AHCC^®^ did not reduce the incidence of chemotherapy-related grade 2–3 anemia compared with placebo (47.3% vs. 44.2%, *p* = 0.84). However, the occurrence of taste disorders was significantly lower in the AHCC^®^ group (23.9% vs. 52.5%, *p* = 0.0077). Patients receiving AHCC^®^ showed improved nutritional parameters, including higher serum albumin, transthyretin, and transferrin, lower CRP, as well as better Mini Nutritional Assessment scores and modified Glasgow Prognostic Score. No differences were observed in tumor response, disease control, or overall survival (median 16 vs. 19 months, *p* = 0.83). Toxicities were mainly related to chemotherapy itself, and AHCC^®^ was well-tolerated without additional safety concerns.	[212] AHCC^®^ did not reduce anemia but significantly lowered chemotherapy-related taste disorders and improved nutritional parameters and quality of life in PDAC patients.
*Lentinula edodes* (Shiitake) Rice bran exo-biopolymer (RBEP, arabinoxylan-rich preparation)	Healthy volunteers–immune modulation, 80 healthy adults (40 RBEP, 37 placebo).	Randomized, double-blind, placebo-controlled, parallel-group clinical trial.	A total of 3 g/day RBEP (6 capsules) for 8 weeks.	↑ IFN-γ secretion (*p* = 0.012 vs. placebo); no effect on NK cell activity or IL-2, IL-4, IL-10, IL-12, TNF-α; no significant adverse effects.	[202] RBEP supplementation is safe and significantly increases IFN-γ levels, suggesting immune-modulatory potential; however, it does not enhance NK activity in healthy individuals.
*Lentinula edodes* (Shiitake) Whole dried shiitake mushrooms	Healthy volunteers–immune modulation, 52 participants randomized (42 completed).	Randomized dietary intervention (parallel groups, no placebo).	A total of 5 g daily *n* = 26; 10 g daily *n* = 25 4 weeks.	Daily intake of dried *Lentinula edodes* for 4 weeks significantly enhanced γδ-T cell proliferation (+60%, *p* < 0.0001) and NK-T cell proliferation (2-fold, *p* < 0.0001), with increased activation markers CD69 and NKG2D. Salivary sIgA rose (*p* ≈ 0.045–0.049) while serum CRP decreased by ~30% (*p* = 0.008). Cytokine shifts included higher IL-4, IL-10, IL-1α, TNF-α and lower MIP-1α, with no changes in IL-1β, IL-6, IL-17, or IFN-γ. The intervention was well-tolerated; only mild gastrointestinal upset and shiitake dermatitis (if undercooked) were reported.	[203] Daily consumption of shiitake mushrooms enhances immune function (γδ-T and NK-T cell activity, sIgA) and reduces inflammation (CRP, MIP-1α).
*Lentinula edodes* (Shiitake) β-d-glucan-enriched (BGE) extract from shiitake	Adults with untreated mild hypercholesterolemia, 52 participants (28 BGE, 24 placebo).	Randomized, double-blind, placebo-controlled, parallel-group clinical trial.	A total of 8 weeks 10.4 g/day mixture providing 3.5 g/day fungal β-d-glucans (incorporated into soups/creams).	BGE was safe and well-tolerated but showed no significant differences in lipid profile (total cholesterol, LDL, HDL, triglycerides) or inflammatory markers (IL-1β, IL-6, TNF-α, oxLDL) compared with placebo. Body weight and BMI slightly decreased within BGE group but not significantly versus placebo. No immunomodulatory effects were observed. However, BGE intake modulated gut microbiota composition differently than placebo, with associations between certain taxa (e.g., *Ruminococcaceae*, *Bifidobacterium*) and cholesterol markers, though clinical relevance was unclear. Main adverse events were mild GI issues (bloating, heartburn, flatulence); dropout ~9%.	[210] BGE extract from shiitake did not lower cholesterol or modify inflammatory markers in mildly hypercholesterolemic adults, but was safe and increased fiber intake. It influenced gut microbiota composition, though clinical significance remains uncertain.
*Lentinula edodes* (Shiitake) Active Hexose Correlated Compound (AHCC^®^), standardized mycelia extract alone or in combination with *Bifidobacterium longum* BB536; placebo control; with/without azithromycin	Healthy young men–immune modulation after antibiotic exposure, 40 men, 18–55 years, 10 per group.	Randomized, double-blind, placebo-controlled trial with 4 groups (placebo, BB536, AHCC^®^, BB536 + AHCC^®^)	A 7-day intervention of AHCC (300 mg/day), *Bifidobacterium longum* BB536 (1.25 × 10^10^ CFU/g), then 5 days of azithromycin 250 mg/day.	Neither AHCC^®^ nor BB536 significantly altered CRP, WBC counts, or T-cell cytokine secretion compared with placebo. BB536 increased Foxp3+ regulatory T cells and the IFN-γ/IL-4 ratio, but this effect was lost after antibiotic exposure. AHCC^®^ alone transiently raised Tregs during the first week, while the combination of BB536 + AHCC^®^ shifted dendritic cell profiles, expanding activated mDC2 with higher CD40 and CD86 expression. All interventions were safe and well-tolerated, with no adverse events reported.	[220] AHCC^®^ and BB536, alone or combined, modulated immune cell phenotypes in healthy men. BB536 and AHCC^®^ increased regulatory T cells, while the combination shifted dendritic cells toward activated mDC2. Effects were modest, transient, and attenuated by antibiotics.
*Lentinula edodes* (Shiitake) Lentinan (β-glucan) + Didanosine (ddI)	HIV-positive patients, CD4 200–500/mm^3^, 107 patients.	Phase II randomized controlled clinical trial.	ddI 400 mg/day for six weeks, followed by the addition of 2 mg of lentinan (i.v.) weekly for 24–80 weeks.	Combination therapy (ddI + lentinan) significantly improved CD4^+^ T-cell counts compared with ddI alone. Mean CD4 increase was sustained until week 38, while in the ddI-only group, the effect plateaued at week 14 and then declined. The combination also reduced opportunistic infection incidence and fatigue scores, and improved immunological markers (↑ CD4/CD8 ratio, ↑ IL-2 production)**.** No serious adverse effects were reported; treatment was well-tolerated.	[204,205] Demonstrated clinically relevant immunomodulatory and adjunctive benefits in HIV therapy; good tolerability profile.
*Grifola frondosa* (Maitake) Polysaccharide extract (D-Fraction)	Patients with breast cancer in remission after completion of therapy, 34 patients	Randomized Phase I/II dose-escalation trial.	Administered doses of 0.1–5 mg/kg twice daily for 3 weeks.	Absence of toxicity; significant, dose-dependent modulation of immune parameters (↑ production of IL-2, IL-10, TNF-α, and IFN-γ by subsets of T cells)	[207] The research provides valuable data on the dose-dependent immunomodulatory effects of maitake, with very good tolerability and no toxicity observed.
*Cordyceps sinensis* (*Paecilomyces hepiali*) Mycelial extract CBG-CS-2	Healthy volunteers—cellular immunity, 79 participants	Randomized, double-blind, placebo-controlled clinical trial.	CBG-CS-2 capsules twice per day (1.68 g/d), 8 weeks.	↑ NK cell activity to 38.8% vs. placebo; increase in the level of IFN-γ of 11.4 ± 7.1%; no adverse effects	[206] The results indicated an increase in NK cell activity, confirming the potential of this preparation as an immunomodulator.
*Cordyceps sinensis* CUF2 formula (capsules of dried aqueous extract with equal parts of 5 herbs: *Astragalus mongholius*, *Cordyceps sinensis*, *Radix stemonae*, *Bulbus fritillariae cirrhosae*, *Radix scutellariae*);	Children with mild–moderate asthma on inhaled corticosteroids, 85 children (42 CUF2, 43 placebo), aged 7–15.	Randomized, double-blind, placebo-controlled trial.	A total of 2–3 capsules twice daily (weight-adjusted); 6 months CUF2 capsule contained 0.619 g of dried aqueous extracts.	Both groups improved, but CUF2 showed no significant benefit over placebo. Steroid dose reduction: −2.3 mg vs. −3.1 mg (*p* = 0.915). Disease Severity Score decreased (−2.3 vs. −3.1, *p* = 0.215). Lung function unchanged (FEV1/FVC +0.1% vs. +0.6%, *p* = 0.809; PEFR −7.3 vs. −0.6 L/min, *p* = 0.118). Biochemical markers (IL-18, TARC, IgE, eosinophils) showed no significant group differences. TNF-α decreased within the CUF2 group (*p* = 0.004), but not vs. placebo (*p* = 0.245). Mild transient adverse events (dry mouth, epistaxis) occurred equally in both groups—no serious adverse effects.	[225] CUF2 (including *Cordyceps sinensis*) was safe but not superior to placebo for reducing steroid use, symptoms, or improving lung function in children with asthma.
*Cordyceps sinensis* Multi-ingredient performance supplement (MIPS, Shroom Tech Sport): Cordyceps extract (1.2 g), Rhodiola extract, Ashwagandha, Astragalus, green tea extract, vitamin B12, chromium.	Active young men undergoing concurrent training, 21 men (10 MIPS, 11 placebo).	Randomized, double-blind, placebo-controlled trial.	A total of 3–5 capsules/day (weight-based); 14 weeks	Both groups improved similarly in % body fat (−1.3%), squat (+8%), and bench (+4%). Cortisol decreased (−11%) without a between-group difference. MIPS group showed higher weekly training workload (bench press volume, total workload, sprint running time at weeks 3–4) but these did not translate into superior overall training outcomes, VO_2_ max, lactate threshold, or body composition compared with placebo. Adverse effects were rare (1 insomnia in MIPS; 2 minor issues in placebo).	[221] MIPS containing *Cordyceps sinensis* and Rhodiola was safe and slightly improved weekly training metrics, but did not enhance overall performance, aerobic capacity, or body composition beyond placebo.
*Cordyceps sinensis* *Grifola frondosa* (Maitake) *Lentinula edodes* (Shiitake) RiteStart^®^ (RS) supplement: multivitamin, multimineral, antioxidants, bovine colostrum/egg yolk transfer factors, omega-3 fatty acids, plus blend including 20 mg *Maitake*, 20 mg *Shiitake*, 74.28 mg *Cordyceps*, olive leaf, inositol hexaphosphate	Healthy adults–overall health, immunity, and nutritional status, 20 enrolled, 13 completed (6 women, 7 men; mean age ~31).	Open-label, uncontrolled pilot intervention trial.	RS twice daily for 12 weeks.	RS significantly increased serum folate (+48%, *p* = 0.0001) and salivary IgA at weeks 4, 8, and 12. Serum vitamin B12 rose by 21% (NS), vitamin D by 9% (NS). RBC slightly decreased, while MCV and RDW increased, suggesting changes in red blood cell parameters. Lipid profile (cholesterol, HDL, LDL, TG) unchanged; fasting glucose rose (78 → 94 mg/dL, *p* < 0.001), coinciding with modest weight/fat gain. SHBG increased, and albumin decreased slightly but within normal range. No significant adverse events; supplement was well-tolerated.	[216] RS supplement was safe, improved folate and sIgA, and may support mucosal immunity, but increased fasting glucose and weight.
*Cordyceps militaris* Functional beverage from submerged fermentation of *C. militaris* (FCM)	Healthy adults–immune modulation, 40 participants (20 men, 20 women; 10 per group).	Randomized, double-blind, placebo-controlled clinical trial.	A total of 75 mL/day for 8 weeks (FCM containing 2.85 mg cordycepin/75 mL).	NK cell activity increased in men at 4 weeks (*p* = 0.049) and in women at 8 weeks (*p* = 0.023) vs. baseline and placebo. Male participants showed reduced IL-1β (*p* = 0.049), females reduced IL-6 (*p* = 0.047), and TNF-α decreased in both sexes. Monocyte counts rose in men at weeks 4 and 8 but remained within normal range. No significant changes in IgA, IgG, IgM, or T/B/NK absolute counts. No differences in metabolic or safety indices; well-tolerated without adverse events.	[208] *Cordyceps militaris* beverage safely enhanced NK cell activity and reduced pro-inflammatory cytokines in healthy adults, supporting potential as an immunostimulatory supplement.

Despite the promising clinical evidence regarding the therapeutic potential of medicinal mushrooms—specifically *Ganoderma lucidum*, *Trametes versicolor*, *Lentinula edodes*, and *Cordyceps* spp.—several critical methodological and technical limitations persist. Most existing literature is based on pilot or Phase I/II trials with small cohorts [201,207,224], which limits statistical power and the generalizability of findings to broader populations. A fundamental challenge remains the lack of extract standardization; studies utilize vastly different substrates, ranging from spore oils and pure mycelium extracts [211,214] to fermented rice bran [202], making it difficult to establish universal dosage protocols. This is further complicated by the frequent failure to distinguish between mycelium-based biomass and pure fruiting bodies, each possessing distinct pharmacological profiles.

Furthermore, discrepancies in extraction methodologies—specifically the choice between hot-water and ethanolic solvents—fundamentally dictate the bioavailability of polar β-glucans versus non-polar triterpenoids, yet these details are often under-reported. Many interventions are also of short duration [208,209], leaving long-term safety and sustained adaptogenic effects unexplored. Additionally, the prevalence of multi-ingredient formulations [216,221] complicates the attribution of clinical outcomes to specific fungal bioactives. Methodologically, the distinct sensory profiles (like characteristic taste or aroma) of fungal extracts pose challenges for effective double-blinding. The sensory recognition of the active treatment may trigger psychological expectancy effects, which potentially overstate the perceived reduction in subjective symptoms such as pain or fatigue [215,223]. Lastly, there is a notable geographical bias, with a heavy concentration of data emerging from Asian cohorts [208,219], which may not fully account for dietary or genetic variations in Western populations. Addressing these analytical, toxicological, and clinical gaps through large-scale, standardized Phase III multicenter trials is essential for the future integration of mycotherapy into evidence-based medicine.

In conclusion, mushroom-derived compounds appear to be safe and well-tolerated in human studies and may provide immunomodulatory benefits as adjunctive therapies, particularly in oncology and immune-related settings. Nevertheless, the heterogeneity of study designs and outcomes, together with the predominance of small or early-phase trials, highlights the need for larger, well-designed randomized controlled studies to establish definitive clinical efficacy and optimal therapeutic indications.

## 5. Limitations and Future Perspectives

Despite the growing body of research on medicinal mushrooms and their bioactive properties, several important limitations should be acknowledged. A significant proportion of the available evidence is derived from in vitro and in vivo studies, while well-designed randomized clinical trials remain relatively limited [227,228]. This discrepancy makes it challenging to directly translate promising preclinical findings into clinical practice. Another important challenge is the lack of standardization of mushroom-derived preparations. The composition of bioactive compounds, such as β-glucans and triterpenoids, may vary considerably depending on species, cultivation conditions, extraction methods, and the part of the mushroom used (e.g., fruiting body or mycelium) [229,230]. Such variability complicates the comparison of results across studies and may affect reproducibility and therapeutic efficacy.

Despite the broad spectrum of biological activities described for the compounds summarized in Table 1, their clinical applicability is often limited by unfavorable pharmacokinetic properties. The bioavailability of many mushroom-derived compounds is not yet fully elucidated. In addition, inconsistencies in production standards and product quality may contribute to variable outcomes, as commercially available preparations from the same mushroom species and strains can differ in composition and effectiveness. Factors such as molecular size, solubility, and interaction with the gut microbiota may influence their absorption and biological activity, which remain incompletely understood [231,232]. Major classes of bioactive molecules identified in medicinal mushrooms, including polysaccharides (e.g., β-glucans from *Lentinula edodes* and *Grifola frondosa*), triterpenoids (e.g., ganoderic acids from *Ganoderma lucidum*), and nucleoside analogs (e.g., cordycepin from *Cordyceps militaris* and *Cordyceps sinensis*), have been reported to exhibit limited aqueous solubility, low intestinal absorption, and susceptibility to metabolic degradation [233,234,235]. These factors are generally associated with reduced systemic exposure and may contribute to decreased therapeutic efficacy. Cordycepin is a well-described example, as it is rapidly metabolized by adenosine deaminase, which results in a short plasma half-life and limited in vivo stability [234,236]. In the case of ganoderic acids, it is suggested that these compounds possess poor aqueous solubility and limited oral bioavailability, which are commonly attributed to their lipophilic nature and metabolic instability. Their high lipophilicity may hinder dissolution and absorption in the gastrointestinal tract and restrict distribution across biological barriers, further contributing to low systemic exposure [237]. These limitations highlight the need for the development of advanced formulation and delivery strategies to improve the pharmacological potential of mushroom-derived compounds. Nanotechnology-based delivery systems, including liposomes, polymeric nanoparticles, and nanoemulsions, have been widely investigated as approaches to improve the solubility, stability, and cellular uptake of poorly water-soluble natural compounds [238,239]. For example, liposomal formulations of *G. lucidum* polysaccharides have been reported to improve formulation stability and biological performance, although challenges such as limited targeting specificity, dose dependence, and relatively short circulation time may still restrict their application. Similarly, nanoparticle-based systems developed for cordycepin have been shown to protect the compound from enzymatic degradation and enable controlled release [233,240]. In addition to nanocarrier systems, inclusion complexation with cyclodextrins has been widely used to enhance the solubility and dissolution behavior of lipophilic compounds, including triterpenoid-rich fractions from *Ganoderma lucidum* and *Trametes versicolor*, as well as nucleoside derivatives from *Cordyceps militaris* [241,242,243,244]. Co-administration with enzyme inhibitors has also been proposed as a strategy to improve the pharmacokinetic profile of specific compounds. In the case of cordycepin, inhibition of adenosine deaminase has been shown to increase its stability and prolong systemic exposure [234,236]. In addition, alternative routes of administration, such as parenteral delivery, may help bypass gastrointestinal limitations and improve systemic availability.

Overall, improving bioavailability remains a key challenge in the translation of mushroom-derived bioactive compounds into clinical applications. The combination of advanced drug delivery systems and pharmacokinetic optimization strategies may enhance their therapeutic potential. However, further preclinical and clinical studies are required to confirm their efficacy and safety and to support the development of standardized formulations derived from medicinal mushrooms [233,235].

Furthermore, many studies report beneficial biological effects without adequately distinguishing between different levels of evidence. The limited differentiation between preclinical and clinical findings may lead to overinterpretation of results and overestimation of therapeutic potential based on non-human data [227,228]. Although adverse effects appear to be relatively uncommon, isolated case reports describing serious neurological complications following multi-component medicinal fungi supplementation indicate that the safety profile of such preparations has not yet been fully elucidated, particularly in pediatric populations [245]. In addition, it should be acknowledged that some of the reported beneficial effects associated with medicinal mushroom preparations, particularly those related to subjective outcomes such as quality of life, mood, fatigue, and general well-being, may be partially influenced by psychosomatic responses, prior experiences, patient expectations, motivational factors, or placebo-related effects rather than exclusively by direct pharmacological mechanisms. Nevertheless, such effects may still contribute to perceived improvements in patient well-being and quality of life, partly explaining the growing popularity of mushroom-derived products within the wellness and nutraceutical sectors and highlighting their potential role in integrative and supportive health approaches [6,246,247].

Considerable uncertainty also exists regarding the appropriate dosage of medicinal mushroom products. The suggested dosages vary widely due to differences in formulations and preparation methods. The absence of well-established standards for recommended doses and duration of administration requires further investigation. While some evidence suggests that very high or very low doses may influence biological responses, these effects have not yet been fully clarified in clinical settings [227,228]. Future research should focus on conducting well-designed, large-scale clinical trials to confirm the efficacy and safety of medicinal mushrooms in humans. Standardization of extracts, including the identification and quantification of key bioactive compounds, is essential to ensure reproducibility and facilitate their application in both nutraceutical and pharmaceutical contexts [229]. Moreover, a better understanding of the mechanisms of action and pharmacokinetic properties of mushroom-derived compounds is needed to support their rational use [231]. Integrating multidisciplinary approaches, including pharmacology, toxicology, and clinical sciences, may further support the development of mushroom-based therapies. Overall, while medicinal mushrooms represent a promising source of bioactive compounds with potential health benefits, further research is required to more clearly establish their clinical relevance and optimize their application in modern biomedical practice.

## 6. Conclusions

Despite their long history of use, medicinal mushrooms remain a relatively unexplored area for many professionals in the medical community; however, this perception is gradually changing. The growing interest in these organisms is driven not only by their deep cultural and historical background but also by the development of modern cultivation techniques and advanced analytical methods that enable the identification of active compounds and their potential synergistic effects.

Numerous studies have demonstrated that medicinal mushrooms possess a wide range of pharmacological properties and are gaining increasing attention as potential therapeutic agents. Several species are already being used, particularly in oncology, for their immunomodulatory and antitumor activities, which may complement conventional treatments, enhance therapeutic efficacy, and potentially reduce side effects. Furthermore, fungi have long served as a valuable source of bioactive compounds—many clinically important drugs, such as antibiotics and the cholesterol-lowering agent lovastatin, are derived from fungal metabolites [248].

Ongoing research aims to elucidate the mechanisms of action of mushroom-derived compounds on the immune system, as well as their potential to inhibit tumor growth or induce tumor cell death. Polysaccharides, especially β-glucans, are among the most extensively studied components and are believed to play a key role in mediating these biological effects.

Mushroom-derived preparations, including *Ganoderma lucidum*, *Trametes versicolor*, *Cordyceps sinensis*, and shiitake, are generally considered safe and well-tolerated in humans. They exhibit potential immunomodulatory effects, such as enhancement of lymphocyte, NK, and T-cell activity, modulation of the gut microbiota, and supportive roles in oncology and immune-related conditions. However, the number of completed human clinical trials remains limited, and not all mushroom species have been adequately investigated. Overall, these compounds may serve as adjunctive therapies, but larger, well-designed randomized trials are needed to confirm their clinical efficacy and establish optimal dosing strategies.

In conclusion, medicinal mushrooms, which have been valued in traditional medicine for centuries, continue to attract scientific attention as a promising yet still underexplored resource for the development of novel functional foods and therapeutic agents, including potential applications in oncology and psychiatric disorders such as depression and PTSD, with increasing interest in psilocybin-containing species. Bridging traditional knowledge with modern analytical and clinical research offers significant opportunities for discovering new bioactive compounds and expanding their safe and effective application in contemporary medicine.

## Figures and Tables

**Table 1:** Bioactive compounds and therapeutic mechanisms of selected medicinal mushrooms.

Mushroom	Selected Active Compounds	Mechanisms of Action	Effects	Ref.
*Trametes versicolor*	Polysaccharide peptide (PSP)	Upregulation of cytokines and chemokines (e.g., TNF-α, interleukins, histamine, PGE); NK cell activation; enhanced dendritic and T cell tumor infiltration in human peripheral blood mononuclear cells Apoptosis induction via mitochondrial pathway: ↓ Bcl-2/Bax ratio, ↓ membrane potential, cytochrome c release, caspase-3/-8/-9 activation in human promyelocytic leukemia HL-60 cells Inhibition of HIV-1 replication; upregulation of antiviral chemokines (RANTES, MIP-1α/β, SDF-1α); coreceptor blockade in THP1 and PBMCs Reduction in cancer cell viability in LoVo, HT-29 cells, HL-60, Hela, and HepG2 cells ↑ glucose transporters in membrane; ↓ glucokinase and glucose-6-phosphatase in liver of T2DM rats	immunomodulatory antitumor, anti-inflammatory, antiviral, liver-protecting, system-balancing, antiulcer, anti-aging, learning and memory-enhancing, antihyperglycemic	[12,13,14,15,16,17,18,19,20,21,22]
	Polysaccharide Krestin (PSK)	↑ DC activation in BALB/c mice; CD4^+^/CD8^+^ T cells; ↓ B cells; ↑ Th1 cytokines secretion, IL-2, IFN-γ; promoted DC maturation (CD86^+^, MHCII); ↑ IL-12p40/p70 in bone marrow derived dendritic cells Activation of cytotoxic T cells; improved DC maturation; ↑ IL-8, TNF-α, IL-1, IL-4, IL-6, IFN-γ via TCR activation; ↑ MHC I expression of tumor cells; ↓ TGF-β; tumor growth inhibition ↓ HIF-1α mRNA → reduction in new blood vessels formation in SW620, HT29 and HCT116 cancer cells ↓ TGF-beta1, uPA, MMP-2, and MMP-9 in human pancreatic cancer cell line NOR-P1 and human gastric cancer cell line MK-1P3 Activation of caspase-3 and induction of p38 MAPK phosphorylation in HL-60 cells ↓ tumor-induced angiogenesis in mice bearing MH134 hepatoma	antitumor, antimetastatic	[15,20,23,24,25,26,27,28,29,30]
	Fruiting body extract	Inhibition of proliferation in T47D, MCF7, and MDA-MB231 cells; induction of apoptosis; ↑ p53; ↓ Bcl-2 Inhibition of migration and invasion; ↓ MMP9 activity and expression in mouse mammary carcinoma 4T1 cells Reduction in colony formation; induction of G0/G1 arrest; promotion of apoptosis; inhibition of invasion and migration; ↓ phospho-PI3K in MCF-7 and HeLa cells ↓ tumor weight and lung metastases in the 4T1-tumor-bearing mouse model ↑ IL-2, IL-6, IL-12, TNF-α, and IFN-γ production in the spleen lymphocytes of tumor-bearing mice	antitumor, antimetastatic, cytotoxic, immunomodulatory	[31,32,33]
	laccase enzyme	Antiproliferative effects on breast (MCF-7), liver (Hep-G2), and cervical (HeLa) cancer cells ↓ cell viability in thyroid (TT) and endometrial (Ishikawa) cancer cells Induction of apoptosis and S-phase cell cycle arrest in Ishikawa cells; ↑ DNA damage in both cancer lines; ↓ Bcl-2; ↑ Bax, Rad51, and ATM expression	cytotoxic, anticancer	[34,35,36]
	triterpenoids	↓ production of NO, TNF-α, and IL-6 in LPS-stimulated RAW264.7 murine macrophages (by eburicoic and trametenolic acid) Inhibition of parasite growth (*Leishmania amazonensis*) by trametenolic acid B	anti-inflammatory	[37,38]
	steroles	↓ production of NO, TNF-α, and IL-6 in LPS-stimulated RAW264.7 murine macrophages (by ergosterol- and lanosterol-derived sterols) Inhibition of parasite growth (*Leishmania amazonensis*) by ergosterol peroxide Bcl-2 inhibition → mitochondrial apoptosis; CDK2/CDK6 inhibition → cell cycle arrest in cancer cell lines (MCF-7, HepG2, A549, HeLa) by ergostanes	anti-inflammatory antiparasitic	[37,38,39]
*Ganoderma lucidum*	triterpenes/triterpenoids, including ganoderic acid T, D, DM	Antimetastatic action via activation of NF-κB and MAP kinase pathways; promotion of cytokine release Induction of apoptosis in metastatic lung cancer cells via mitochondrial dysfunction pathway and ↑ p53 expression ↓ ALT/AST; ↓ oxidative stress, ↓ NF-κB in mice → hepatocyte protection Inhibition of proliferation in HeLa carcinoma cells; induction of G2/M cell cycle arrest and apoptosis by ganoderic acid D Arrest of osteoclastogenesis in bone marrow and RAWD cells by ganoderic acid DM via suppression of c-Fos and NFATc1; inhibition of DC-STAMP expression; reduction in osteoclast fusion ↑ expression of osteoblast differentiation markers: RUNX2, OSX, OPN, ALP, osteocalcin (OCN), and COL1α1; signaling cross-talk between Wnt/β-catenin and BMP/SMAD → pro-osteogenic effect of Ganoderal A in human amniotic mesenchymal stem cells (hAMSCs) ↓ secretion of TNF-α, IL-6, inflammatory mediator NO and prostaglandin E_2_ (PGE_2_) from lipopolysaccharide (LPS)-stimulated murine RAW264.7 cells → anti-inflammatory effects mediated by the inhibition of transcription factor NF-κB Downregulation of expression cyclin D1, CDK4, and cyclin B1 → ↓ cell proliferation of RAW264.7 cells through cell cycle arrest at G0/G1–G2M	anticancer, hypoglycemic, immunomodulatory, antihypertensive, cytotoxic, antidiabetic, antioxidant, antihyperlipidemic, antimicrobial, hepatoprotective, osteoprotective	[40,41,42,43,44,45,46,47,48,49,50,51,52,53,54,55]
	polysaccharides	Inhibition of hepatic stellate cell (HSC) and activation of collagen production in mice and in TGF-β1-induced HSC-T6 cells Inhibition of spontaneous and Fas-induced apoptosis in human neutrophils via activation of PI3K/Akt pathway and prevention of procaspase-3 degradation; reversible by PI3K inhibitors Induction of cell cycle arrest and apoptosis in human myeloid leukemia HL-60 cells via ↓ ERK, ↑ p38, and ↑ JNK; regulation of p53, Bax/Bcl-2, and caspase-3 Activation of death receptor Fas and caspase-8 leading to apoptotic cell death; ↑ intracellular Ca^2+^ and ↑ LDH levels; inhibition of migration of HCT-116 human colon cancer cells ↑ cell-surface expression of CD80, CD86, CD83, CD40, CD54, and human leukocyte antigen (HLA)-DR, ↑ production of interleukin (IL)-12p70, p40, and IL-10, and also IL-12p35, p40, and IL-10 mRNA expression in human monocyte-derived dendritic cells (DC) → induction of activation and maturation of human DC by the NF-κB and p38 MAPK pathways ↓ expression of pro-inflammatory cytokines (TNF-α, IL-6, IL-1β, and interferon (IFN)-γ in the colon of female C57BL/6 mice ↓ glucose, ↓ lipid peroxidation, PI3K/Akt modulation → improved insulin sensitivity in type 2 diabetic mellitus rats Promotion of wound healing via activation of the Wnt/β-catenin pathway and ↑ TGF-β1 in primary human skin fibroblasts and in the Kunming mouse model	hepatoprotective wound healing, anti-fibrotic, anticancer, immunomodulatory	[42,43,44,45,46,47,56,57]
	fruiting body extract	Significant inhibition of viability in tumor cell lines (A549, SW1990, SKOV3, HCT116); HCT116 cells more sensitive to GLE (IC50 = 106 µg/mL); induction of apoptosis via ↓ Bcl-2/Bax ratio, ↑ cleaved caspase-3, and ↑ PARP expression; increased autophagy via autophagosome formation and altered mTOR pathway proteins; G0/G1 cell cycle arrest; ↓ tumor weight and volume; ↓ Ki67 expression in vivo Potential antitumor effects against kidney 786-O and liver HepG2/C3A cancer cells; exhibiting cytotoxic and genotoxic activities at low concentrations ↑ SOD, CAT, GPx → ↓ ROS, ↓ lipid peroxidation; ↓ IL-6, ↓ TNF-α in male BALB/c mice	antitumor, genotoxic, anti-aging	[58,59,60]
*Lentinula edodes*	polysaccharides	Inhibition of proliferation in MCF-7 cells via ↑ p53 expression and suppression of HER-3 activity in a dose- and time-dependent manner Inhibition of proliferation of Sarcoma 180 (S-180) solid tumor cells and human colorectal cancer cell lines (HT-29 and HCT-116) in vitro (5 mg/mL) Reduction in the infectivity of viruses: adenovirus (Ad7), herpes simplex 2 (HSV-2), and SARS-CoV-2 Inhibitory effect on HIV-1 reverse transcriptase activity, reduction in leukemia cell proliferation (L1210) reduction in blood glucose levels, ↓ triglycerides, and cholesterol in rats receiving a high-fat diet (HFD) Suppression of pro-inflammatory cytokine expression and colitis in mice Amelioration of synaptic ultrastructure alteration, neuroinflammation, and BDNF deficits induced by HF diet via β-glucan supplementation; ↑ mucosal thickness; ↑ occludin expression; ↓ plasma LPS levels; inhibition of pro-inflammatory macrophage accumulation in colon ↑ circulating monocytes and CD8^+^ T cells; ↓ CD4/CD8 ratio; reduction in IL-4, IL-6, IL-10, and GM-CSF production by lentinan supplementation in rat models	anti-colitis, anti-inflammatory, immunomodulatory, neuroprotective, cytotoxic, anticancer, antiviral, antihypertensive, antidiabetic, cardioprotective	[56,61,62,63,64,65,66,67,68,69,70,71]
*Grifola frondosa*	D-fraction	Activation of macrophages, T cells, and NK cells; ↑ expression of BAK-1 and genes (ST7, RASSF-2, FADD, ITGA2, IGFBP-7, ICAM3, SOD2, Cul-3, CAV-1, NRF2, Cyclin E, SPARC) involved in apoptosis stimulation, inhibition of cell growth and proliferation, cell cycle arrest, suppression of tumor migration and metastasis in MCF-7 breast cancer cells ↓ PI3K-AKT signaling pathway in HT-29 cell line → induction of apoptosis ↑ E-cadherin levels, inhibition of MMP-2 activity in MDA-MB-231 cell line → induction of apoptosis; reduction in cell motility and invasiveness; ↑ ALP activity, calcium deposition → promotion of osteoblast differentiation in human mesenchymal stem cells (hMSCs)	antitumor, immunostimulatory, pro-osteogenic	[72,73,74,75,76]
	polysaccharides	Upregulation of NO and TNF-α through the TLR-4 in RAW264.7 cells → improvement of immune functions Hypoglycemic effects via enhanced insulin sensitivity; ↑ IR and IRS-1 activities; ↓ fasting serum glucose (FSG), fasting serum insulin (FSI), and HOMA-IR; ↑ protein levels of IR; ↓ IRS-1 protein levels; activation of PI3K/Akt pathway; ↑ mRNA levels of PI3K and Akt in diabetic rat model Upregulation of JAK2/STAT3/SOCS signaling pathway → protection against cyclophosphamide-induced immunosuppression in mice Amelioration of Alzheimer’s-like pathology and cognitive impairments via enhanced microglial amyloid-β clearance in APP/PS1 transgenic mice ↑ SOD, CAT, GPx → antioxidant properties in high-fat diet–streptozotocin (STZ)-induced type 2 diabetic rat model ↓ TNF-α, IL-2 → anti-inflammatory effect in high-fat diet–streptozotocin (STZ)-induced type 2 diabetic rat model Indirect cytotoxic activity against HepG-2 cells, inhibition of the growth of Heps cells in vivo in mice	hypoglycemic, neuroprotective, immunomodulatory, antioxidant, antitumor	[77,78,79,80,81]
*Inonotus obliquus*	polysaccharides	↓ proliferation of SW620 colorectal cancer cells ↑ cell viability; ↓ apoptosis and caspase-3 activity; ↓ LDH release; restoration of mitochondrial membrane potential; ↓ intracellular ROS in L-Glu-damaged HT22 cells Nrf2 pathway activation and upregulation of HO-1, SOD, and CAT in UVB-induced HaCaT keratinocytes ↑ glycogen storage in liver; ↑ glucose uptake; ↑ PI3K-p85, p-Akt (Ser473) and GLUT4 protein levels in STZ-induced type 2 diabetic mice → improvement of insulin resistance Stimulation of the immune system ↑ expression of NLRP3inflammasome, IL-1β, and IL-18 in colitis-associated cancer model of mice ↑ HO-1, ↑ SOD, ↑ Nrf2 expression in the brain of APP/PS1 transgenic mice → protection from neuronal oxidative stress via activation of the Nrf2/HO-1 pathway	hepatoprotective, antiobesity, anticancer, antidiabetic, anti-inflammatory, neuroprotective	[19,82,83,84,85,86,87,88]
	triterpenoids	Inhibition of NO production and pro-inflammatory enzymes (iNOS, NF-κB) by lanostane triterpenoids (inonotusols, trametenolic acid) in the BV-2 microglial cell line; ↑ cytotoxicity of trametenolic acid on human prostatic carcinoma cell PC3 and breast carcinoma MDA-MB-231 cell line Inhibition of α-glucosidase in HepG2 cells via activation of PI3K/Akt and GLUT4 pathways by inotodiol ↑ GSH content and SOD and CAT activities, ↓ MDA content; activated Nrf2 signaling, ↑ expression of HO-1 and NQO-1; ↓ NF-κB signaling and TNF-α, IL-6, and IL-1β expressions in a spontaneous diabetic nephropathy model of C57BLKS/db (db/db) mice (by trametenolic acid) ↓ xanthine oxidase (XO) activity; ↓ ROS and inflammatory mediators → ↓ hyperuricemia and inflammation in hyperuricemic mice by triterpenoid acids	anti-inflammatory, anticancer, antidiabetic, renoprotective	[89,90,91,92]
	steroids	↑ cytotoxicity of ergosterol peroxide on human prostatic carcinoma cell PC3 and breast carcinoma MDA-MB-231 cell line ↓ NF-κB activity, ↓ iNOS expression, ↓ NO production in LPS-stimulated RAW264.7 macrophages by ergosterol peroxide ↓ proliferation and colony formation in HCT116, HT-29, SW620 and DLD-1 CRC cell lines ↓ nuclear levels of β-catenin → ↓ transcription of c-Myc, cyclin D1, and CDK-8 by ergosterol peroxide ↑ ROS → mitochondrial membrane potential (MMP) and significant ↓ of cellular respiration and glycolysis in TNBC models ↓ tumor volume and metastases to the lungs and liver in vivo in SHO-SCID mice inoculated with MDA-MB-231-GFP cells (by ergosterol peroxide)	Anticancer Anti-inflammatory	[91,93,94]
*Cordyceps militaris*	polysaccharides	Activation of RAW 264.7 macrophages (↑ phagocytosis, ↑ NO production); ↑ mRNA expression of pro-inflammatory cytokines (TNF-α, IL-6) via the MAPK pathway ↑ protein and mRNA expression level of caspase-3, caspase-9, and p53, ↓ protein and mRNA expression levels of proliferating cell nuclear antigen (PCNA) → induction of H1299 lung cancer cells apoptosis ↑ phagocytosis function and ↑ M1 polarization of RAW 264.7 macrophages; upregulation of T-cell population → via TLR2, MAPK, NF- κB pathways ↑ production of short-chain fatty acids (SCFAs), ↑ beneficial bacterial groups (Actinomycetota and Bacillota),↓ potentially harmful bacterial groups (Pseudomonadota and Fusobacteriota) Activation of Nrf2/HO-1 and NF-κB pathways; ↑ HO-1 SOD enzymes in an ovalbumin-induced allergic asthma mouse model ↓ glucose levels, ↓ serum lipids, and improved intestinal dysbiosis through promoting the population of next-generation probiotic *Akkermansia muciniphila* in the gut of mice fed HFSD Improved glucose metabolism, serum lipid profiles, hormone secretion, and gut microbiota composition in T2DM mouse models	antitumor, antioxidant, immunostimulatory, antimicrobial, prebiotic, hepatoprotective, neuroprotective, hypoglycemic	[95,96,97,98,99,100,101,102,103,104,105,106]
	cordycepin	Cytotoxic activity, suppression of early apoptosis, increased late apoptosis/necrosis in SNU719 cells; suppression of EBV transfer from LCL-EBV-GFP cells to human gastric adenocarcinoma (AGS) cells Inhibition of SARS-CoV-2 replication and blocking of viral genome transcription/replication in Vero E6 cells High binding affinity to viral proteins (RdRp, spike protein, and Mpro) in silico → may support antireplicative potential against SARS-CoV-2 Inhibition of NF-κB, iNOS, COX-2; ↓ NO and ↓ PGE_2_ in RAW 264.7 macrophage cells ↓ NO and PGE_2_ induced by IL-1β in human osteoarthritis chondrocytes ↓ NO, PGE_2_, TNF-α, and IL-1β; ↓ NF-κB, Akt, MAPKs in BV-2 murine microglial cells Inhibition of adipogenesis and lipid deposition in adipocytes in in vitro experiments (3T3-L1 pre-adipocytes) via suppression of the C/EBPβ, PPARγ, and mTORC1 pathways and activation of AMPK Activation of Wnt/β-catenin signaling,↑ runt-related transcription factor 2 (RUNX2), collagen 1, osteoprotegerin (OPG);↓ receptor activator of nuclear factor-B ligand (RANKL), and oxidative stress → promotion of osteogenesis and inhibition of osteoclastogenesis in human bone marrow mesenchymal stem cells (BM-MSCs) and ovariectomized (OVX) and aged mouse models ↓ triglycerides, total cholesterol, LDL, and VLDL levels in hamsters and rats fed with a high-fat diet ↓ expression of ICAM-1, IL-4, IL-5, IL-13, and eosinophils in BAL fluid, ↓ NF-κB signaling pathway activation in the Ova-driven asthmatic mice, suppression of IgE in mice serum → reduction in airway hyperresponsiveness	antiviral, immunomodulatory, anti-inflammatory, antihyperlipidemic, antiasthmatic, osteoprotective	[100,107,108,109,110,111,112,113,114,115,116,117]
*Cordyceps sinensis*	polysaccharides	Free radical scavenging (DPPH, OH^−^, O_2_^−^), metal chelation chemical radical assays in vitro Activation of macrophages and lymphocytes; modulation of cytokines (↑ IL-1β, ↑ IL-6, ↑ IL-12, ↑ TNF-α, ↑ IFN-γ); RAW 264.7 macrophage polarization shift M2 → M1 via NF-κB Induction of apoptosis via caspase activation (↑ caspase-3, -9, ↑ Bax/Bcl-2 ratio, ↑ cytochrome c) in HepG2 cells Modulation of gut microbiota: ↑ probiotics (*Lactobacillus*, *Bifidobacterium*, *Bacteroides*); ↓ pathogenic bacteria (*Clostridium*, *Flexispira*) ↑ SCFA levels; support of intestinal immunoregulation on cyclophosphamide (Cy)-induced intestinal mucosal immunosuppression and microbial dysbiosis in BALB/c mice ↓ hepatic glucose and glucose transporter type 2 (GLUT2) levels in (STZ)-induced diabetic rats and epinephrine-induced hyperglycemic mice ↑ NO; ↓ ET-1, ↓ epinephrine, ↓ noradrenaline, ↓ angiotensin II; ↓ TGF-β1 and ↓ CRP in spontaneously hypertensive rats (SHR) → improved vascular function and reduced hypertension ↑ SOD, CAT, GPx activities; ↓ lipid peroxidation in in vivo animal models	immunomodulatory, anticancer, antihypertensive, hepatoprotective, neuroprotective, prebiotic	[118,119,120,121,122,123,124,125,126,127,128,129,130,131]
	cordycepin	Caspase activation, ↑ p53, activation of A3 adenosine receptor, cyclin D1 suppression, inhibition of tumor cell proliferation and migration Inhibition of platelet aggregation, ↓ MMP-2/MMP-9 activity, ↑ TIMP-1/2 → ↓ cancer cell invasiveness in LNCaP human prostate carcinoma cells ↓ ROS generation, ↓ cytotoxicity induced by palmitic acid, ↓ inflammatory mediators in human vascular endothelial cells (HUVECs) → protective changes in vascular endothelial cells Antidepressant-like effect via modulation of gut microbiota in C57BL/6 J mice: ↑ SCFA production, ↓ IL-1β, ↓ IFN-γ → suppression of systemic inflammation	antitumor, antioxidant, anti-atherosclerotic, antidepressant	[132,133,134,135,136,137]

Abbreviations: ALT—alanine aminotransferase; AST—aspartate aminotransferase; ATM—ataxia telangiectasia mutated (serine/threonine protein kinase); BAK-1—BCL2 antagonist/killer 1; BDNF—brain-derived neurotrophic factor; Bcl-2—B-cell lymphoma 2 protein; C/EBPβ—CCAAT/enhancer-binding protein beta; CAT—catalase; COX-2—cyclooxygenase-2; CRP—C-reactive protein; DC—dendritic cell; DC-STAMP—dendritic cell-specific transmembrane protein; DPPH—2,2-diphenyl-1-picrylhydrazyl; EBV—Epstein–Barr virus; ePSP—extracellular polysaccharopeptides; ERK—extracellular signal-regulated kinase; ET-1—endothelin-1; GM-CSF—granulocyte–macrophage colony-stimulating factor; GPx—glutathione peroxidase; HCMP—hepatic cholesterol metabolism parameter; HER-3—human epidermal growth factor receptor 3 (ErbB3); HFD—high-fat diet; HO-1—heme oxygenase-1; HOMA-IR—homeostatic model assessment of insulin resistance; IFN-γ—interferon gamma; iNOS—inducible nitric oxide synthase; IR—insulin receptor; IRS-1—insulin receptor substrate 1; JNK—c-Jun N-terminal kinase; Ki67—Ki-67 proliferation antigen; LDL—low-density lipoprotein; LPS—lipopolysaccharide; MAP kinase—mitogen-activated protein kinase; MHCII—major histocompatibility complex class II; MIP-1α/β—macrophage inflammatory protein 1 alpha/beta (CCL3/CCL4); MMP-9—matrix metalloproteinase 9; NF-κB—nuclear factor kappa B; NFATc1—nuclear factor of activated T cells, cytoplasmic 1; NK cell—natural killer cell; NLRP3—NOD-like receptor family pyrin domain-containing 3; NO—nitric oxide; Nrf2/HO-1—nuclear factor erythroid 2–related factor 2/heme oxygenase-1 signaling pathway; PARP—poly(ADP-ribose) polymerase; PGE—prostaglandin E; PI3K/Akt—phosphoinositide 3-kinase/protein kinase B signaling pathway; PPARγ—peroxisome proliferator-activated receptor gamma; PSK—polysaccharide-K (krestin); PSP—polysaccharopeptide; Rad51—RAD51 recombinase; ROS—reactive oxygen species; SCFA—short-chain fatty acids; SDF-1α—stromal cell-derived factor 1 alpha (CXCL12); SOD—superoxide dismutase; STZ—streptozotocin; T2DM—type 2 diabetes mellitus; T47D—human breast carcinoma cell line; TCR—T-cell receptor; TG—triglycerides; TGF-β1—transforming growth factor beta 1; TIMP-1—tissue inhibitor of metalloproteinases 1; TLR-4—toll-like receptor 4; TNF-α—tumor necrosis factor alpha; VLDL—very low-density lipoprotein; SNU719 cells—EBV genome-integrated gastric carcinoma cell line; LCL—Lymphoblastoid Cell Line; CDK-8—Cyclin-dependent kinase-8.

## Data Availability

No new data were created or analyzed in this study. Data sharing is not applicable to this article.
